# A micronucleus assay detects genotoxic effects of herbicide exposure in a protected butterfly species

**DOI:** 10.1007/s10646-020-02276-3

**Published:** 2020-09-03

**Authors:** Alfredo Santovito, Michela Audisio, Simona Bonelli

**Affiliations:** Department of Life Sciences and Systems Biology, University of Turin, Via Accademia Albertina 13, 10123 Torino, Italy

**Keywords:** Pesticides, Roundup, Genomic damage, Lepidoptera, Habitats directive

## Abstract

*Lycaena dispar* Hawort (Lepidoptera: Lycaenidae), a protected butterfly, is declining in Europe, but it thrives in rice fields in northern Italy. Here, agrochemical usage could threaten its long-term survival. We investigated, by micronucleus (MN) assay, the genotoxic effect of glyphosate, a common herbicide, on *L. dispar* larvae. Micronuclei (MNi) are DNA fragments separated from the main nucleus and represent the result of genomic damage that has been transmitted to daughter cells. In a control/treatment experiment, we extracted epithelial cells from last-instar larvae fed with *Rumex* spp. plants sprayed with a solution containing 3.6 g/L of glyphosate, and from larvae fed with unsprayed plants. MNi and other chromosomal aberrations—nuclear buds (NBUDs) and bi-nucleated cells—were then scored in 1000 cells/subject. Significant differences were found between glyphosate-exposed and control groups in terms of MNi and total genomic damage, but not in terms of NBUDs or bi-nucleated cells. We reported a possible genomic damage induced by glyphosate on larvae of *L. dispar*. For the first time, a MN assay was used in order to evaluate the genomic damage on a phytophagous invertebrate at the larval stage. Increased levels of MNi reflect a condition of genomic instability that can result in reduced vitality and in an increased risk of local extinction. Therefore, farmland management compatible with wildlife conservation is needed.

## Introduction

In the last decades, the expansion of intensive agriculture has triggered serious ecological problems, in terms of loss of habitats and biodiversity. This is principally due to the excessive use of pesticides, which control populations of targeted pests, but also reduce the fitness of non-target species (Siroski et al. 2016). Pesticides directly and indirectly affect wildlife (Isenring 2010). The use of insecticides and herbicides, for instance, was found to negatively impact butterfly abundance and species richness (for a review see Braak et al. 2018). Indeed, herbicide treatments lead to a reduction of native plants and the insect communities they support; in some cases, this results in a reduction in population size and a higher risk of extinction (Thogmartin et al. 2017; Stenoien et al. 2018). Many studies ascribe the decline of animal populations to agrochemical treatments, for example, birds (Mineau and Whiteside 2013; Donald et al. 2001), reptiles (Mingo et al. 2016; Wagner et al. 2015), amphibians (Brühl et al. 2013; Wagner et al. 2014), and bats (Stahlschmidt and Brühl 2012). Recently, the EU Pollinators Initiative named pesticide use as one of the main causes of the loss of managed bee colonies and the decline of wild bees and other insect pollinators (EU 2019). Although the drivers of this phenomenon are not well understood, there is increasing evidence of negative effects of pesticides on wild and managed bees (Sánchez-Bayo et al. 2016), bumblebees (Stanley et al. 2016) and butterflies (Forister et al. 2016).

In Europe, the mosaic of habitats resulting from traditional farm management has favored a high diversity of flora and fauna (Bignal and McCracken 2000). Agroecosystems host more than half of the 29 European protected butterfly species (van Swaay et al. 2012), and are crucial for threatened butterflies and wild bees (van Swaay et al. 2010; Nieto et al. 2014). Pesticide treatments and other activities connected to agricultural intensification can be a threat to these vulnerable species. For example, pesticides have been reported as one of the causes of the steady decline of some butterflies listed in the Habitats Directive 92/43/EEC (1992) by the European Economic Community (EEC) observed from 1960 (van Swaay et al. 2012). In Italy, 92% of protected habitats and 56% of protected animal species are vulnerable to pesticides. Of these, wetlands and their species are the most sensitive (ISPRA 2015). Artificial water surfaces, such as rice fields, can act as surrogate habitats for wetland species (Fasola and Ruiz 1996; Bambaradeniya et al. 2004; Giuliano and Bogliani 2018).

In the 1900s, *Lycaena dispar* Hawort (Lepidoptera: Lycaenidae), a butterfly listed in the Annexes II–IV of the Directive 92/43/EEC (1992), has disappeared from many natural and semi-natural wetlands (Pullin et al. 1995; Balletto et al. 2005). At the same time, in the River Po region of northern Italy, *L. dispar* has adapted to live in rice fields, and has actually increased in number as a result of rice cultivation (Balletto et al. 2005; Bonelli et al. 2011). Vegetated rice fields banks are, in fact, where the butterfly finds larval food plants (belonging to the genus *Rumex*) and nectar sources. In rice crops, field banks are usually intensively managed, to remove weeds with chemical or mechanical procedures (Cardarelli and Bogliani 2014). Intensive management practices, such as the use of non-selective herbicides, have turned the rice field in an ‘ecological trap’, which has led to a rapid population decline of *L. dispar* in the second half of the 1900s. (Balletto et al. 2005; Bonelli et al. 2018). In particular, it has been demonstrated that glyphosate spraying, by directly removing food sources, drastically reduces the presence of *L. dispar* (Giuliano et al. 2018). Moreover, we hypothesize that chronic exposure to herbicides can determine sublethal effects that may, in the long term, reduce the fitness and have evolutionary implications. In Italy, rice fields are mainly located in Piedmont, and here the mean annual sales of glyphosate accounted for 3915 tons from 2009 to 2012 (ISPRA 2017).

Glyphosate (N-phosphonomethyl glycine) is a widely used, systemic non-selective herbicide (Costa et al. 2014; Tarazona et al. 2017). Despite its reputation of being safe, glyphosate can contaminate soil and water, leading to cascading effects on non-target organisms (Helander et al. 2012). Toxic effects of glyphosate have been reported for a wide range of organisms (Gill et al. 2018); for example, it was found to cause behavioral and physiological alterations in vertebrate (Avigliano et al. 2014; Navarro-Martín et al. 2014; Uren Webster et al. 2014; Hansen and Roslev 2016) and invertebrate species (Schneider et al. 2009; Balbuena et al. 2015; Xu et al. 2017; Blot et al. 2019). We also have evidence of genotoxicity of this chemical; for instance, in human lymphocytes it was found to increase the frequency of micronuclei (MNi) (Santovito et al. 2018), whereas other forms of DNA damage have been described in different cell types (Monroy et al. 2005; Mañas et al. 2009; Koller et al. 2012). However, commercial pesticide products comprise the active substance with adjuvants or surfactants. Although these last are considered inert ingredients, in many cases their toxicity exceeds or intensifies the toxicity of the active ingredient (Beggel et al. 2010; Cox and Surgan 2006; Beggel et al. 2010). For these reasons, recently, the cytotoxic and genotoxic effects were also evaluated on glyphosate-based herbicides, such as Roundup®. Roundup® has been found able to cause genomic damage to human peripheral blood mononuclear cells at a concentration of 5 μM, a value lower than that observed for glyphosate, which was found to induce DNA lesions starting from a concentration of 500 μM (Woźniak et al. 2018). The genotoxic effect of glyphosate-based herbicides has already been tested with MN assay on some vertebrate taxa, such as fish (Grisolia 2002; Cavalcante et al. 2008), reptiles (Poletta et al. 2009; Schaumburg et al. 2016), and amphibians (Bosch et al. 2011). On the other hand, nobody tested the response of phytophagous terrestrial invertebrates, although they can be exposed to this herbicide through their diet.

To reduce pesticide risk and impact on human health, as well as the environment and biodiversity, is an issue of major concern in the European union (Council Directive 2009/128/EC 2009). For this reason, to decrease the overall use of chemical pesticides is one of the aims of the EU Biodiversity Strategy for 2030 (EC 2020a). Protecting the environment is also a goal of the Regulation (EC) 1107/2009 (2009) for plant-protection-products (PPP) authorization. In order to authorize a new PPP, standard toxicity tests are required using specific test subjects. However, an open challenge is to develop a risk assessment scheme to specifically address indirect impacts of pesticides on different organisms, since no guidelines are presently available (Streloke 2011). Specifically, to better understand the impacts of agrochemicals, more ecotoxicological data are required to assess sublethal effects of a chemical on non-target species, as well as those of conservation concern. There is very little evidence about the impact of agrochemicals on protected species; for example, the bioaccumulation of organoclorurate pesticides in the Eurasian otter (*Lutra lutra* Linnaeus) (Lemarchand et al. 2010). In fact, nowadays laboratory toxicity tests use a limited number of test species and only examine lethal effects over short time frames, while ecologically relevant sublethal effects are less frequently described. Many of the tests use insensitive species and are not sufficiently long to represent chronic exposure and, therefore, lack environmental relevance.

In the light of what have been said, the aim of the present work was to investigate—by micronucleus (MN) assay—the genotoxic effect of the glyphosate-based herbicide Roundup on larvae of *L. dispar*. MNi represent chromosome fragments or whole chromosomes that fail to segregate properly during mitosis, appearing in interphase as small additional nuclei. They are the result of clastogenic or aneugenic damage that has been transmitted to daughter cells. Chromosomal instability was also measured by scoring both NBUDs, which represent the elimination process of amplified DNA and/or excess chromosomes from aneuploid cells, and bi-nucleated cells, that are the result of impairment of cytodieresis (Fenech et al. 2011). The tested hypothesis was to evaluate if Roundup® affects *L. dispar* in terms of genomic damage induction at the larval stage, with consequent reduction in larvae vitality and with important evolutionary implications.

## Materials and methods

### Larval rearing experiment and exposure to glyphosate

*Rumex* leaves with *L. dispar* eggs were collected in a rice field in Rovasenda (Vercelli, Piedmont), 45°33′01″ N, 8°19′25″ E, at the beginning of September 2019. We decided to collect the eggs in a place where they would have ended up in a ‘ecological trap’ due to management practices, so we selected a field margin that was going to be moved in a few days, compromising the survival of the larvae. The sampling of the eggs of this protected species was authorized by the Italian Ministry of the Environment, Land and Sea, by derogation from Article 16 of the Directive 92/43/EEC (1992) (protocol number 26142). Eggs were then brought to the laboratory to be raised. A total of 85 eggs were collected, and then placed in Petri dishes in groups of ten. Fresh *Rumex* leaves were added every day for the newly-hatched larvae. At day 1 after hatching, the young larvae were divided in two groups; one group was placed on a *Rumex* plant sprayed with 1% Roundup® Power 2.0 containing 3.6 g/L of glyphosate, which represents a dose commonly used in agricultural practices (glyphosate-exposed group), and the other on an unsprayed *Rumex* plant (control group). Plants and larvae were kept in two separated net-cages in a climate cell at 26 °C L:D 15:9 (typical environmental conditions in their natural habitat during summer) and the plants were watered every 2–3 days. Sprayed plants withered after 8–9 days so they were replaced with new fresh plants, and sprayed with the herbicide, in order to provide larvae with food.

### Micronucleus assay

The larvae reached the last instar after 15–18 days, and were then dissected; their cuticle was cut using a micro-scissor and the hemolymph was collected by rubbing on a slide, while the epithelial cells were collected using a spatula. The tip of the spatula was directly dipped into a tube containing a fixative solution of 3:1 methanol/acetic acid and stored at 4 °C prior to analysis. Successively, cells were collected by centrifugation, supernatants were discarded and the pellets, dissolved in a minimal volume of fixative, were seeded onto slides to detect MNi by conventional staining with 5% Giemsa (pH 6.8) prepared in Sörensen buffer. Microscopic analysis was performed at ×400 magnification on a light microscope, whereas checking and photos of micronucleated cells were performed at ×1000 magnification. MNi, NBUDs, binucleated and karyolitic cells were scored in 1000 cells per subject, following the established criteria for MNi evaluation (Thomas et al. 2009).

### Statistical analysis

Statistical analyses were conducted using the SPSS software statistical package program (version 25.0, IBM Corp 2017). Differences between glyphosate-exposed and controls were evaluated by Kruskal–Wallis non-parametric test. All *P*-values were two-tailed and the level of statistical significance was set at *P* < 0.05 for all tests.

## Results

We obtained 64 larvae from the eggs that we collected, which were divided into 32 glyphosate-exposed and 32 control subjects. Development time of the larvae was comparable between the two groups (about two weeks). A sample of 6 glyphosate-exposed larvae and 13 control individuals was randomly chosen and allowed to pupate, and they reached the adult stage without any visible morphological anomalies.

**Fig. 1:**
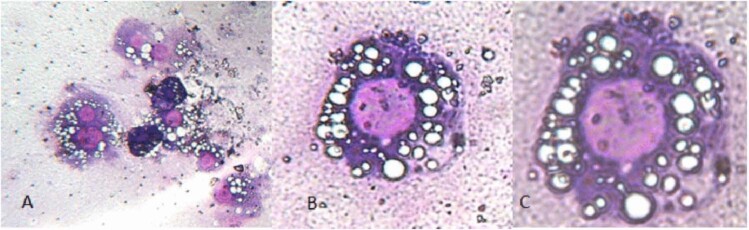
Examples of haemocytes stained with 5% Giemsa observed in both control and glyphosate-exposed samples. **a** Haemocytes observed at ×160 magnification. **b**, **c** Haemocytes observed at ×400 and ×1000 magnification, respectively

**Fig. 2:**
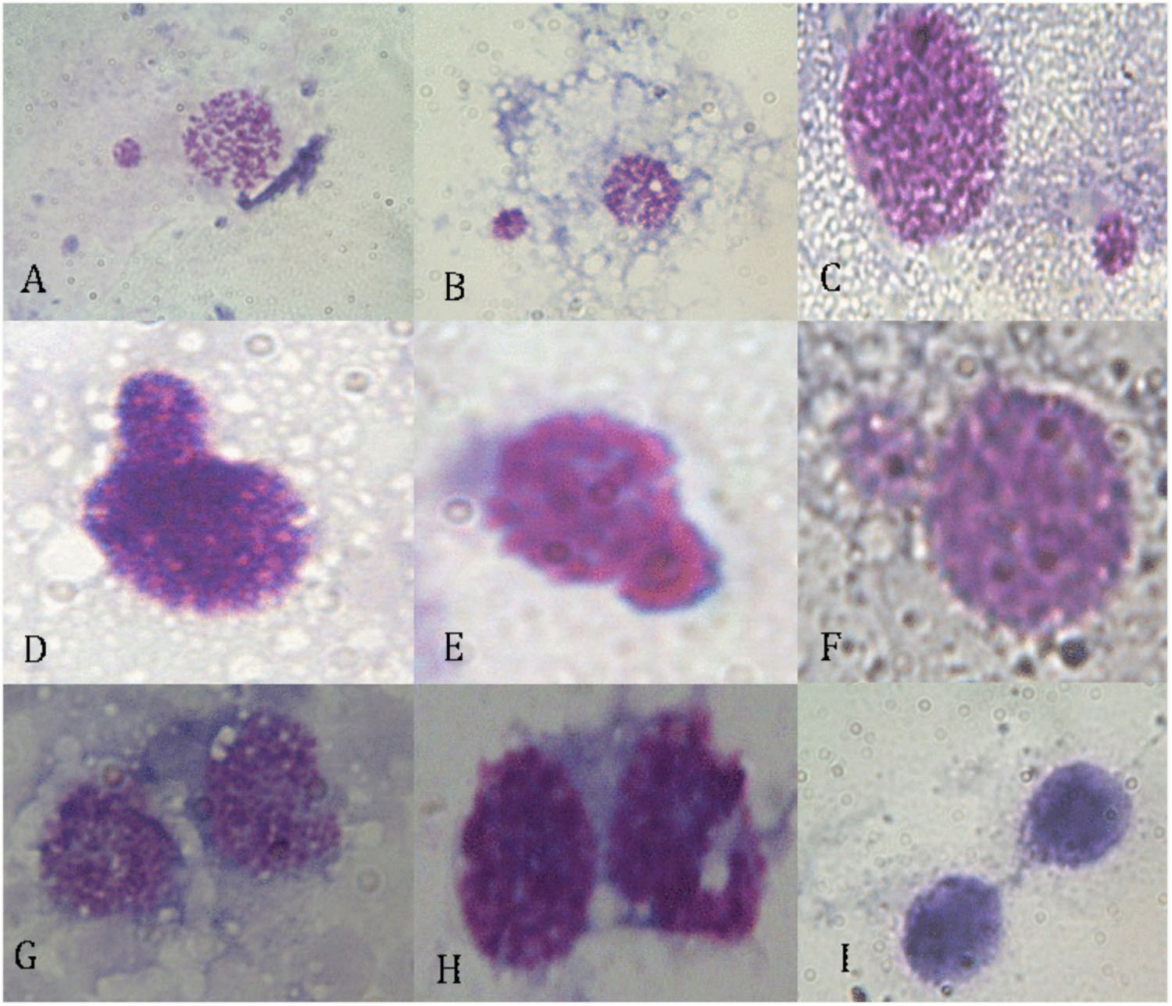
Examples of micronuclei and nuclear buds observed in the samples, stained with 5% Giemsa and observed at ×400 (**a**, **b**, **i**) and ×1000 magnification (**c**, **d**, **e**, **f**, **g**, **h**). **a**–**c** Cells with micronucleus; **d**–**f** nuclear buds; **g**–**i** bi-nucleated cells

Finally, a MN assay was performed on 26 glyphosate-exposed larvae and 19 control subjects. Although only about 200 cells were scored for six subjects (three controls and three glyphosate-exposed larvae), we decided to include them in the analyzed sample. A total of 16,585 and 23,757 cells were analyzed for control and glyphosate-exposed larvae, respectively. In Fig. 1, some examples of observed haemocytes in both control and exposed larvae are reported. Unfortunately, it was not possible to apply the MN assay to haemocytes due to their intense cytoplasmic vacuolization, which interferes with the observation of MNi and NBDUs. In Table 1, the frequencies of MNi, NBUDs and bi-nucleated cells in both glyphosate-exposed and control groups are reported. In Fig. 2, some examples of the observed damaged cells are shown. In the control group, the frequency of MNi, NBUDs and bi-nucleated cells were 0.137 ± 0.164, 0.066 ± 0.121, and 0.126 ± 0.148 respectively, whereas in the glyphosate-exposed group, these frequencies were 0.442 ± 0.337, 0.177 ± 0.127 and 0.204 ± 0.328, respectively. Significant differences were found between glyphosate-exposed and control groups in terms of MNi (H = 9.974, *P* = 0.002) and total genomic damage (H = 11.996, *P* = 0.001), whereas the differences in NBUDs (H = 3.427, *P* = 0.064) and bi-nucleated cells (H = 0.044, *P* = 0.883) frequencies were not significant, although the glyphosate-exposed group showed higher values (0.117 ± 0.127 and 0.204 ± 0.328 respectively).

**Table 1:** Results of the statistical evaluation of genomic damage between glyphosate-exposed and control groups. Only significant results were reported

	*N*	Cells	MNi (mean ± SD%)	NBUDs (mean ± SD%)	Bi-nucleated cells (mean ± SD%)	Total genomic damage (mean ± SD%)
Controls	19	16,585	26 (0.137 ± 0.164)	9 (0.066 ± 0.121)	24 (0.126 ± 0.148)	35 (0.203 ± 0,171)
Glyphosate-exposed	26	23,757	102 (0.442 ± 0.337)*	27 (0.117 ± 0.127)	44 (0.204 ± 0.328)	129 (0.560 ± 0.420)**

Only significant results were reported

*MNi* micronuclei, *NBUDs* nuclear buds, *SD* standard deviation

*P = 0.002; **P = 0.001, compared to the controls (Kruskal–Wallis test)

## Discussion

Many authors have pointed out that excessive pesticide usage has a negative effect on wildlife, especially on birds (Donald et al. 2001; Mineau and Whiteside 2013), reptiles (Mingo et al. 2016), amphibians (Brühl et al. 2013), bats (Stebbings and Griffith 1986) and insect pollinators (Forister et al. 2016; Sánchez-Bayo et al. 2016; Stanley et al. 2016).

Glyphosate is the most widely used broad-spectrum herbicide in the world, with known toxicity (Avigliano et al. 2014; Navarro-Martín et al. 2014; Uren Webster et al. 2014; Zhang et al. 2019). However, glyphosate-based herbicides, as well as all other pesticides, contains inert ingredients that facilitate the access to cells by the active component but that, in some cases, can harm humans and the environment (Giesy et al. 2000; Cox and Surgan 2006). In this sense, new testing methodologies and new ecotoxicological studies on commercial glyphosate-based formulations are needed to provide information about their toxicity to non-target organisms (van der Sluijs et al. 2015). The toxicity of glyphosate commercial formulations has been well studied across vertebrates, such as fishes, amphibians and reptiles (Navarro-Martín et al. 2014; Uren Webster et al. 2014; Carpenter et al. 2016; Siroski et al. 2016). In this article, we have shown that sublethal exposure to glyphosate-based herbicide Roundup® is able to induce, in *L. dispar* larvae, a significantly increased level of genomic damage, in terms of a higher frequency of MNi.

Sublethal effects of both glyphosate and glyphosate-based herbicides on invertebrates’ development have been already documented. For example, bee larvae fed with food containing glyphosate traces (1.25–5.0 mg per litre of food) were shown to have delayed molting and reduced weight (Vázquez et al. 2018). Similarly, in damselfly larvae, Roundup concentrations from 1.5 to 2 mg/l were found to reduce growth and to increase the expression levels of the stress protein Hsp70 (Janssens and Stoks 2017). Sublethal doses of pesticides are also well known to impact insect behaviors. For example, sublethal exposure to deltamethrin was found to diminish maternal egg care (Meunier et al. 2020), to impair orientation and memory and to increase stimulus sensitivity, general excitement and disordered movements (Zhang et al. 2020; Decourtye et al. 2004; Ramirez-Romero et al. 2005; Thompson 2003). However, whereas understanding of sublethal effects typically focuses on physiological and behavioral parameters (Mc Luckie et al. 2020; Meunier et al. 2020), in the present work we emphasize the importance of sublethal effects on genome integrity and, more importantly, we revealed for the first time that these effects can be observed also at the larval stage. Indeed, here we report a possible genomic damage induced by Roundup, a common glyphosate-based herbicide applied in a broad diversity of crops worldwide, on larvae of *L. dispar*, a butterfly listed in the Directive 92/43/EEC (1992). In particular, for the first time, the MN assay was used in order to evaluate the genomic damage on a phytophagous invertebrate at the larval stage. Although MN assay requires the dissection of the studied animal, the positive aspect is that it allows the observation, in a short time and on a non-target organism, of biological effects due to chronic exposure to sublethal doses of an herbicide. Moreover, it should be recalled that these non-lethal effects are not commonly assessed in regular exposure impact studies. This work also provides important insights on the diversity of sublethal effects possibly occurring in insects, and confirming the potentiality of the use of non-model organisms to study them.

Increased levels of MNi reflect a condition of genomic instability that can result in a reduction of vitality and an increased extinction risk. Indeed, MNi do not only are the product of cytogenetic errors, but, recently, it has been suggested that they represent a mechanism of elimination of genetic material, such as amplified genes, and that they could contribute to nuclear dynamics and genome chaos (Heng 2019; Ye et al. 2019). Genome chaos represents a process of rapid genome re-organization that results in the formation of chaotic genomes, some of which could be selected to establish stable genomes (Ye et al. 2019). Not all MNi can be simply eliminated, but some of them may perform biological functions, such as DNA synthesis, and/or rejoining into other nuclei, further contributing to abnormal karyotypes or changing the coding genome by integrating new genomic material (Ye et al. 2019). It seems that the stress response is the key evolutionary mechanism in terms of short-term adaptation. Pesticides may act as stressors, and, in this sense, genome chaos could represent an evolutionary mechanism that provides a survival strategy under stress, by means of genomic re-organization. Interestingly, genome chaos has also been hypothesized to represent an effective means for speciation, as it could be responsible for a rapid speciation event during massive extinctions (Heng 2019; Ye et al. 2019). This could be the case of the Italian population of *L. dispar*; in rice fields, agrochemicals and shortage of food sources can act as strong selective pressures. As was demonstrated by Gibbs and Van Dyck (2010) on the butterfly *Pararge aegeria* Linnaeus, rapid changes in life history traits occur in highly fragmented and low habitat-quality human-modified landscapes. Thus, in this anthropic environment, positive selection for individuals with a higher mobility and with greater fecundity is predicted. However, this is a hypothetical and optimistic scenario, and, actually, for the studied species, we have no evidence of a successful adaptation process. *Vice versa*, it is more likely that the effects inducing genomic chaos may lead to a decreased average fitness and to an associated increased risk of local extinction.

## Conclusions

In the face of the dramatic disappearance of natural wetlands, rice fields are a perfect example of a surrogate wetland habitat for *L. dispar*, as well as other wetland species (Pullin et al. 1995; Balletto et al. 2005), but pesticides (in particular herbicides) can compromise their long-term survival, not only by change in habitat quality and depletion of food sources but also by genotoxic effects.

In view of these detrimental effects on a European protected species, crop protection strategies compatible with wildlife conservation are needed. In particular, as stated by the Regulation (EC) 1107/2009 (2009), it is desirable to reduce pesticide input and have a correct use of chemicals when and where they are needed. In the case of Roundup, the latter aspect is not entirely negligible, given that very often, in common agricultural practices, it is over-sprayed without precautions for avoiding drift spray (low-drift and low-pressure injectors) and without respecting buffer zones (Cederlund 2017).

Finally, it is necessary to give priority to non-chemical and natural alternatives, for example to promote organic farming; this is actually the aim of the EU Pollinator Initiative and the focus of the debate for the next Common Agricultural Policy reform (EC 2020b).

## Data Availability

The authors declare the availability of data and material.

## Legend

**Aneugenic****damage**: Damage caused by a substance (aneugen) that causes a daughter cell to have an abnormal number of chromosomes.
**Aneuploid cells**: Cells with an abnormal number of chromosomes, typically with 1 or more missing or excess chromosomes.
**Binucleated cells**: Cells with two nuclei instead of one. The significance of these cells is unknown but they may be indicative of failed cytodieresis following the nuclear division.
**Cytodieresis**: The division of the cytoplasm of a cell following the division of the nucleus.
**Clastogenic damage**: Damage caused by a mutagenic agent (clastogen) inducing disruption or breakages of one or more chromosomes.
**EEC**: European Economic Community.
**ISPRA**: Istituto Superiore per la Protezione e la Ricerca Ambientale, Italian name of the Italian Institute for Environmental Protection and Research.
**Karyolytic cells**: Cells with a nucleus completely depleted of DNA and that apparent as a ghost-like image. These cells thus appear to have no nucleus. They represent a very late stage in the cell death process.
**MN**: Micronucleus. It represents a chromosome fragment or whole chromosomes that fails to segregate properly during cell division cycle, appearing in interphase as small additional nuclei.
**MNi**: Micronuclei. Plural of Micronucleus.
**NBUDs**: Nuclear buds. They represent the physical manifestation of the elimination process of excess amplified DNA.
**PPP**: Plant-protection products.
